# Iodized Salt Consumption in Sudan: Present Status and Future Directions

**Published:** 2012-12

**Authors:** Mohamed Salih Mahfouz, Abdelrahim Mutwakel Gaffar, Ibrahim Ahmed Bani

**Affiliations:** ^1^Family and Community Medicine Department, Faculty of Medicine, Jazan University, Saudi Arabia;; ^2^Population Studies Center, Gezira University, Sudan;; ^3^Member of the International Council for the Control of Iodine Deficiency Disorders (ICCIDD)

**Keywords:** Iodine deficiency disorders, Salt legislations, Universal Salt Iodization, Sudan

## Abstract

Iodine Deficiency Disorders (IDD) Control Programme in Sudan adopted salt iodization as the long-term strategy in 1994. In 2000, it was found that less than 1% of households were using adequately-iodized salt. The objectives of this study were to: (i) study the coverage and variation of different geographical regions of Sudan regarding access to and use of iodized salt, (ii) explore the possible factors which influence the use of iodized salt, (iii) develop recommendations to help in the implementation of the Universal Salt Iodization (USI) strategy in Sudan. This paper is based on the Sudan Household Health Survey (SHHS) dataset. A total sample of 24,507 households was surveyed, and 18,786 cooking salt samples were tested for iodine levels with rapid salt-testing kits. Nationally, the percentage of households using adequately-iodized salt increased from less than 1% in 2000 to 14.4%, with wide variations between states. Access to iodized salt ranged from 96.9% in Central Equatoria to 0.4% in Gezira state. Population coverage with iodized salt in Sudan remains very low. The awareness and political support for USI programme is very weak. National legislation banning the sale of non-iodized salt does not exist. Utilization of the already-existing laws, like the National Standardization and Metrology Law (2008), to develop a compulsory national salt specification, will accelerate the USI in Sudan.

## INTRODUCTION

Iodine is an essential element for growth and development of humans and animals because it is a constituent of the thyroid hormones which play a crucial role in metabolism. The term ‘iodine deficiency disorders’ (IDD) refers to all the effects of iodine deficiency on growth and development in human and animal population (1). IDD increases the risk of cretinism, stillbirth, miscarriage, and infant mortality. In developing countries, about 38 million newborns every year are unprotected from the lifelong consequences of brain damage associated with iodine deficiency disorders (2). Elimination of iodine deficiency partly contributes to achieving the Millennium Development Goals agreed upon by UN Member States in 2000. Meeting these goals would transform the lives of millions of children and families (2).

Universal Salt Iodization (USI) strategy to ensure sufficient intake of iodine by all individuals was recommended by the WHO and UNICEF Joint Committee on Health Policy in 1994 (3). Salt iodization is a remarkably cost-effective public-health strategy. On average, the one-time increase in cost is only 3-5 cents per person per year—a price so low that even consumers in the least developed countries would barely notice it (4). There are two forms of iodine in iodized salt: ‘iodide’ and ‘iodate’, usually as the potassium salt. Both are generally referred to as ‘iodized’ salt (3,5). Iodate is recommended as the preferred fortificant because it is much more stable. Nearly 70% of the salts for humans and livestock in the world are iodized today (4). UNICEF estimates that less than 20% of households in the developing world were using iodized salt in the early 1990s, and by 2000, the average had jumped to 70% (2). By 2006, around 120 countries were implementing salt iodization programmes (2).

Worldwide, 34 countries have eliminated iodine deficiency disorders through Universal Salt Iodization (USI). Throughout the Middle East and North Africa region, 64% of households consume adequately-iodized salt, with wide variation between countries. The Islamic Republic of Iran, Lebanon, and Tunisia are considered to have achieved the goal of Universal Salt Iodization (6). In Algeria, Egypt, Jordan, the Occupied Palestinian Territory, Oman, and the Syrian Arab Republic, household consumption of adequately-iodized salt is at least 50%. In Sudan, Iraq, and Yemen, the consumption of adequately-iodized salt is extremely low (2,6).

IDD Control Programme in Sudan started in October 1989, using Lipiodol in some of the highly-endemic regions of the country and adopted salt iodization as the long-term national strategy in 1994 (7). By 2000, only 0.6% households were consuming adequately-iodized salt in Northern Sudan while in Southern Sudan, it was less than 0.5%. The percentage of households using adequately-iodized salt ranged from 0.1% in River Nile state to 3.6% in South Darfur state. One percent of urban households were using adequately-iodized salt compared to 0.4% of rural households (8). A comprehensive study conducted in 2006 showed that the goitre prevalence (GP) in school children aged 6 to 12 years in Sudan was found to be 38.8% overall, ranging from 12.2% in Omdurman to 77.7% in Kosti city (9). The urgent need for and the progress toward Univesrasl Salt Iodization in different Sudanese states were felt and discussed in many regional studies (10,11,12).

The objectives of this study are to: (i) study the coverage of and variation in different geographical parts of Sudan regarding access to and use of iodized salt, (ii) explore the possible factors which influence the use of iodized salt, and (iii) develop recommendations to help in the implementation of the USI strategy in Sudan.

## MATERIALS AND METHODS

Sudan is the largest country in Africa with an area of about 2.5 million kilometres. It neighbours nine countries with wide-open borders. The population in Sudan was estimated at 39.2 million in 2008, and the majority (72%) lives in rural areas (13).

This study was based on the dataset of the Sudan Household Health Survey (SHHS) conducted in 2006. The survey was implemented by the Federal Ministry of Health (FMoH), the Central Bureau of Statistics (CBS), and the Ministry of Health (MoH), together with the Southern Sudan Commission for Census Statistics and Evaluation (SSCCSE) (14).

The SHHS provides valuable information on the health situation of households, children, and women in Sudan. The survey was initiated, in a large part, on the need to have a baseline national data to monitor progress toward goals and targets emanating from national plans and international agreements—the Millennium Development Goals (MDGs). The sample for SHHS was designed to provide estimates on some key indicators of the health situation of children and women at the national level and in the 25 states of Sudan. In each state, a sample of 1,000 households was drawn for the survey.

The household questionnaire covered a number of topics, such as education, water and sanitation, household income and resources, use of insecticide-treated nets, salt iodization, and maternal mortality.

Of the 24,527 households initially selected as the sample, 24,507 households were found to be occupied. Of these, 24,046 households were successfully interviewed for a household response rate of 98.1%. During the SHHS, 18,786 cooking salt samples were tested for iodine levels (eg. presence of potassium iodate) with rapid salt-testing kits (RTK), using colour reference indicator.

The Statistical Package for Social Science (SPSS, version 17) was used for analyzing the data. General tabulations including frequency distribution were used. Chi-square test was used for testing some associations between the dependent variable (iodized salt) and a set of independent variables. The iodized salt test variables were recoded into two codes only, either using iodized salt (positive test) or not using iodized salt. In each case, a test of independence was performed using chi-square test of independence. The independent variables that assumed to affect the use of iodized salt (dependent variable) are: education of the head of the household, gender, mode of living, the region, and wealth index.

## RESULTS

Table 1 illustrates the regional distribution of households according to the use of iodized salt and adequacy of salt iodization. One in seven households (14.4%) in Sudan was using adequately-iodized salt, i.e. >15 parts per million. Twenty percent of urban population and 11.7% of the rural population are using adequately-iodized salt. The percentage of household using adequately-iodized salt ranged from 50% in Equatoria region to less than 0.5% in Northern region. People's access to iodized salt ranged from 88% in regional sector to 1% in the central region (Table 1).

**Table 1.** Geographical distribution of households according to the use and adequacy of salt iodization in Sudan

Category	Salt test result*			Total No. (%)
	Not iodized No. (%)	<15ppm No. (%)	>15ppm No. (%)	
Region				
Northern	1,926 (98.5)	23 (1.2)	7 (0.4)	1,956 (100)
Eastern	2,733 (95.0)	66 (2.3)	77 (2.7)	2,876 (100)
Khartoum	937 (98.2)	8 (0.8)	9 (0.9)	954 (100)
Central	3,813 (98.9)	21 (0.5)	21 (0.5)	3,855 (100)
Kordofan	1,714 (91.9)	66 (3.5)	85 (4.6)	1,865 (100)
Darfur	1,812 (63.0)	196 (6.8)	866 (30.1)	2,874 (100)
Upper Nile	430 (52.1)	318 (38.5)	77 (9.3)	825 (100)
Bahar-Algazal	509 (34.0)	473 (31.6)	517 (34.5)	1,499 (100)
Equatoria	240 (11.5)	799 (38.4)	1,043 (50.1)	2,082 (100)
Mode of Living				
Urban	4,344 (72.6)	439 (7.3)	1,198 (20.0)	5,981 (100)
Rural	9,770 (76.3)	1,531 (12.0)	1,504 (11.7)	12,805 (100)
Total	14,114 (75.1)	1,970 (10.5)	2,702 (14.4)	18,786 (100)

*Not iodized: no colour change;

<15 ppm (parts per million): slight blue colour change;

>15 ppm: deep blue colour change

Table 2 as well as the Figure classify the Sudanese states into four groups, based on the percentage of households having access to iodized salts as recommended in the WHO/UNICEF 2007 joint statement. It is clear that only one state has more than 90% of households with access to iodized salt in 7 states, 50-90% of households have access to iodized salt; in 5 states, 20-50% of households have access to iodized salt, and in 12 states, less than 20% of households have access to iodized salt. The percentage of households consuming adequately-iodized salt ranged from 0.2% in Northern state to 78.9% in Central Equatoria state. Only three states (Central Equatoria, Lakes, and East Equatoria) had more than 50% of households using adequately-iodized salt.

**Table 2.** Classification of states according to the percentage of households having access to iodized salt

Group	State
Group 1: More than 90% of the households having access to iodized salt	Only one state: Central Equatoria (iodized—96.9%; adequately iodized—78.9%)
Group 2: 50-90% of the households having access to iodized salt	7 states: Upper Nile (iodized—87.4%, adequately iodized—14.6%); Lakes (iodized—84.6%, adequately iodized—59.3%); West Equatoria (iodized—81.4%, adequately iodized—13.4%); East Equatoria (iodized—81.1%, adequately iodized—50.5%); Warab (iodized—77.3%, adequately iodized—11.7%); Unity (iodized—58.3%, adequately iodized—10.8%); West Bahr Al-Gazal (iodized—57.7%, adequately iodized—31.4%)
Group 3: 20-50% of the households having access to iodized salt	5 states: West Darfur (44.1%), Northern Darfur (44.0%), Northern Bahr Al-Gazal (39.7%), Jongolei (26.1%), South Darfur (23.0%)
Group 4: Less than 20% of the households having access to iodized salt	12 states: Northern Kordofan (8.2%), Red Sea (8.0%), South Kordofan (8.0%), Gadarif (4.4%), Kassala (2.6%), River Nile (2.0%), Khartoum (1.8%), White Nile (1.5%), Sinnar (1.5%), Northern state (1.0%), Blue Nile (1.0%), Gezira (0.4%)

**Figure.**
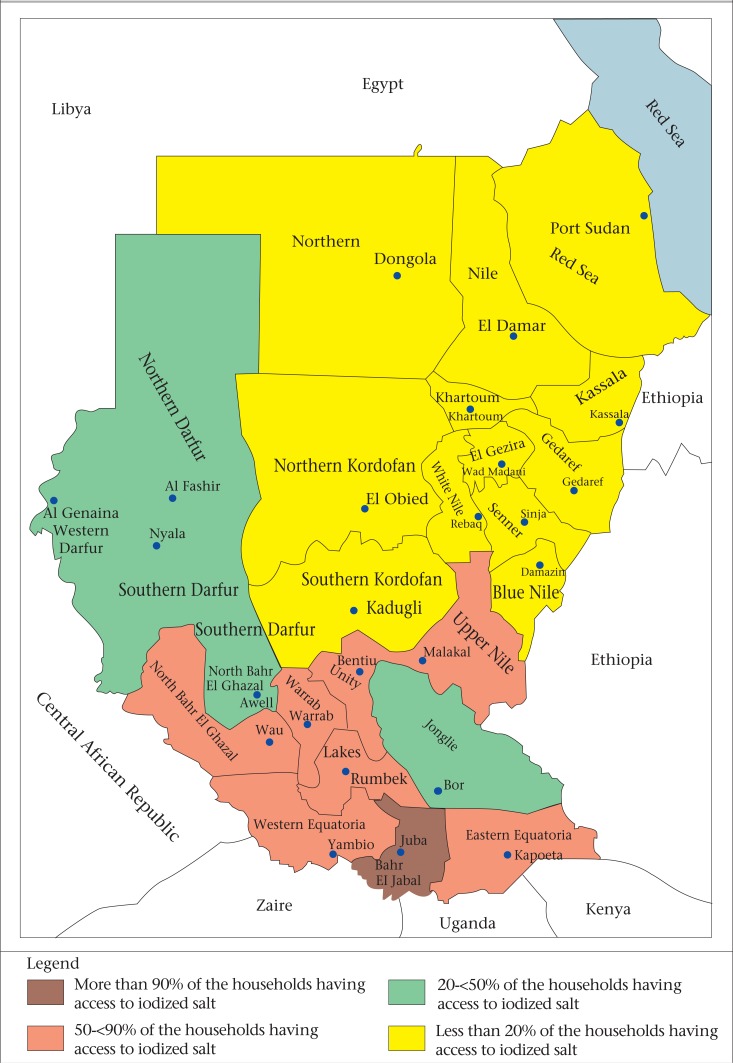
Map showing the use of iodized salt in Sudan

Table 3 shows the adequacy of salt iodization according to the source of the salt by geographical area. In general, around 90% of the survey population obtained their salt from the local markets. In Darfur (28.58%), the salt is provided by Food Aid. Nationally, only 21.2% of salts from the local markets are iodized while 89.37% of the salts from Food Aid are iodized; only 10.72% of salts from the local markets are adequately-iodized compared to 64.0% of the salts from Food Aid. The availability of iodized salt in the markets ranged from around 90% in Equatoria sector to less than 0.5% in Northern sector (Table 3).

**Table 3.** Adequacy of salt iodization according to the source of the salt by geographical area

Sectors test results*	Source of salt							
	Local market—No. (%)			Total	Food Aid—No. (%)			Total
	Not iodized	<15 pmm	>15 pmm		Not iodized	<15 pmm	>15 pmm	
Northern	1,901 (98.9)	15 (0.8)	7 (0.4)	1,923 (100.0)	3 (33.3)	6 (66.7)	0 (0.0)	9 (100.0)
Eastern	2,543 (96.9)	43 (1.6)	39 (1.5)	2,625 (100.0)	19 (32.2)	16 (27.1)	24 (40.7)	59 (100.0)
Khartoum	928 (98.5)	6 (0.6)	8 (0.8)	942 (100.0)	1 (33.3)	2 (66.7)	0 (0.0%)	3 (100.0)
Central	3,508 (99.1)	14 (0.4)	17 (0.5)	3,539 (100.0)	10 (66.7)	5 (33.3)	0 (0.0)	15 (100.0)
Kordofan	1,668 (93.8)	47 (2.6)	64 (3.6)	1,779 (100.0)	7 (17.1)	17 (41.5)	17 (41.5)	41 (100.0)
Darfur	1,773 (87.7)	83 (4.1)	165 (8.2)	2,021 (100.0)	26 (3.2)	112 (13.6)	685 (83.2)	823 (100.0)
Upper Nile	395 (58.6)	232 (34.4)	47 (7.0)	674 (100.0)	14 (12.6)	73 (65.8)	24 (21.6)	111 (100.0)
Bahar-Al-Gazal	446 (34.8)	398 (31.1)	437 (34.1)	1,281 (100.0)	44 (25.3)	63 (36.2)	67 (38.5)	174 (100.0)
Equatoria	216 (10.9)	755 (38.0)	1,014 (51.1)	1,985 (100.0)	17 (20.0)	40 (47.1)	28 (32.9)	85 (100.0)
Total	13,378 (79.8)	1,593 (9.5)	1,798 (10.7)	16,769 (100.0)	141 (10.7)	334 (25.3)	845 (64.0)	1,320 (100.0)

*Not iodized: no colour change

<15 ppm (parts per million): slight blue colour change

>15 ppm: deep blue colour change

Table 4 shows association between iodized salt-use and some assumed independent variables. The table shows that educational level of the head of the household, gender, mode of living, the region, and wealth index have strong association with the consumption of iodized salt. Households are more likely to consume iodized salt if the head of household is male, lives in urban setting or live in the areas where iodized salt is accessible in the markets. On the other hand, households are more likely to consume iodized salt if the head of household is more educated and comparatively wealthy (Table 4).

**Table 4.** Association between the use of iodized salt and some selected factors

Variable	Using iodized salt No. (%)	Not using iodized salt No. (%)	Chi-square value and p value
Education of the household head			
No education	6,627 (67.9)	3,127 (32.1)	χ^2^=627.658 p=0.000
Primary	3,093 (80.2)	765 (19.8)	
Secondary	2,896 (82.8)	603 (17.2)	
Gender of the household head			
Male	12,282 (77.7)	3,531 (22.3)	χ^2^=344.985 p=0.000
Female	1,832 (61.6)	1,141 (38.4)	
Mode of living			
Rural	4,344 (72.6)	1,637 (27.4)	χ^2^=29.361 p=0.000
Urban	9,770 (76.3)	3,035 (23.7)	
Region			
Northern Sudan	12,935 (90.0)	1,445 (10.0)	χ^2^=7207.985 p=0.000
Southern Sudan	1,179 (26.8)	3,227 (73.2)	
Wealth index			
Poorest	1,831 (52.2)	1,680 (47.8)	χ^2^=2659.936 p=0.000
Second	2,633 (61.8)	1,626 (38.2)	
Middle	3,282 (77.9)	933 (22.1)	
Fourth	3,619 (93.2)	264 (6.8)	
Richest	2,749 (94.2)	169 (5.8)	

## DISCUSSION

At the national level, there is an overall improvement in the percentage of households using adequately-iodized salt from less than 1% in 2000 to 14.4% in 2006 (8) but this is still a very low coverage for USI goal. UNICEF estimates that the average in developing countries had reached 70% in 2000 (2). Among the countries neighbouring Sudan, the lowest coverage of households with adequately-iodized salt is found in Ethiopia and Chad, which have 20% and 56% coverage respectively. Kenya, Libyan Arab Jamahiriya, and Uganda achieved the goal of Universal Salt Iodization. Congo and Egypt are in progress to achieve this goal (6).

There are wide variations in people's access to iodized salt among the states, ranging from 96.9% in Central Equatoria to 0.4% in Gezira state. It is the first time ever since the inception of the programme in Sudan to report these high levels of access to iodized salt in some states. Similar variations were reported in other countries, like Bangladesh and India (15,16). The implementation of the salt iodization programmes varies between states, thus resulting in different levels in people's access to iodized salt. Based on the UNICEF/WHO joint statement and classification (17), the programme priority interventions in the state level must be based on the situation of the USI programme in each state; this will enhance the utilization in federal system and the autonomous level of governance.

The variation in iodized salt consumption is largely due to the great variation among states regarding the availability of iodized salt in the markets. In Equatoria region, 89.1% of the salts in the local markets are iodized, and 51.1% are adequately iodized. On the other hand, 98.9% of the salts in the local markets in the Northern region are not iodized. The main improvement occured in Southern and Darfur states. Markedly significant changes observed in southern states are likely due to the improvement in the security situation as a result of the Comprehensive Peace Agreement (CPA) signed by the Government of Sudan and the Sudan People's Liberation Movement (SPLM) in 2005 (12). Salts in these states mainly come from Kenya and Uganda across the borders, and the percentage of households using adequately-iodized salt in Kenya and Uganda is 91% and 96% respectively (6,8). On the other hand, the coverage in the Darfur states is high because Food Aid, in recent years, increased distribution in the region in response to the civil conflict and humanitarian crisis. Generally, salts from Food Aid are more likely to be iodized compared to only the fifth of the salt samples available in the local markets.

The main source of salt in other parts of Sudan is in the Red Sea state, and a small quantity of iodized salt is imported from Saudi Arabia, Turkey, and countries in the Middle East (10). The iodine levels are not at the range recommended by the Sudan Ministry of Health, thus need to be monitored at entry-port to ensure adherence to the national specification. If production of iodized salt is ensured from all local producers, the majority of population will have access to iodized salt.

Although there is considerable international support to the National USI programme, especially in the form of donation of salt iodization machines and potassium iodate, iodized salt production is stagnant (18). This reflects the low awareness and weak political commitment toward USI and IDD control (18,19). Many ministerial decrees were issued by ministers of health, requesting salt manufacturers to produce iodized salt (18), in addition to legislations banning the sale of non-iodized salt in three states. However, the implementation and enforcement of legislations are of concern. Instead of wasting IDD/USI programme resources to pass new legislations for banning non-iodized salt in this non-supportive political environment, the programme can use the existing laws and legislations to enforce USI strategy. The programme should work with Sudan Standardization and Metrology Organization (SSMO) and Consumer Protection Organization to issue compulsory salt specification for the nation and develop monitoring system, based on the National Standardization and Metrology Law 2008 (20) and other laws, like National Public Health Law (2008) (21), Food Inspection Law (1973) (22), National Council for Childhood Care Law (2008) (23), and School Health Law (1974) (24) .

All examined factors, such as education of the head of the household, gender, mode of living, the regions, and wealth index, are significant at p<0.05 in bivariate analysis. This means that all these variables are strongly associated with iodized salt-use. This reflects clearly that Universal Salt Iodization is a public-health policy issue rather than individual choice. Whenever and wherever iodized salt alone is available at the markets, people will use it even though they are not aware of its benefits.

The IDD programme has no monitoring and evaluation system; monitoring of IDD prevention and control programmes is crucial to ensure that additional iodine intake is effective in reducing the deficiency while preventing excessive intake that may lead to adverse health consequences (3). The only national IDD survey was conducted in 1997; there is an urgent need to conduct national IDD survey to update the country data, using the WHO/UNICEF/ICCIDD guidelines and to establish monitoring systems.

### Limitations

Using only rapid salt-testing kits to determine iodine content was one of the limitations of the study. This method needs to be supported by titration method, using the standard WHO/UNICEF/ICCIDD guidelines. The other limitation was that we could not access the Multiple Indicators Cluster Survey (MICS, 2000) datasets to re-analyze it as we did with SHHS data; this was important since it was possible to explore the trend in population coverage with iodized salt.

### Conclusions

The population coverage with iodized salt in Sudan remains very low. The awareness and political support for the USI programme is very weak. National legislation banning non-iodized salt does not exist. Utilization of already-existing laws, like the National Standardization and Metrology Law (2008), to develop a compulsory national salt specification will accelerate the USI in Sudan. Establishment of monitoring systems, including monitoring the market availability of iodized salt, is crucial. National IDD survey is urgently needed to update the country data and for future planning.

## ACKNOWLEDGEMENTS

We thank the Sudan Household Health Survey, 2006 coordinating authority who gave us the survey data and all dedicated data-collection workers who participated in this national survey.
